# Friend or Foe: S100 Proteins in Cancer

**DOI:** 10.3390/cancers12082037

**Published:** 2020-07-24

**Authors:** Chantal Allgöwer, Anna-Laura Kretz, Silvia von Karstedt, Mathias Wittau, Doris Henne-Bruns, Johannes Lemke

**Affiliations:** 1 Department of General and Visceral Surgery, Ulm University Hospital, Albert-Einstein-Allee 23, 89081 Ulm, Germany; chantal.allgoewer@uni-ulm.de (C.A.); anna-laura.kretz@uni-ulm.de (A.-L.K.); mathias.wittau@uniklinik-ulm.de (M.W.); sekretariat.chirurgie1@uniklinik-ulm.de (D.H.-B.); 2 Department of Translational Genomics, Center of Integrated Oncology Cologne-Bonn, Medical Faculty, University Hospital Cologne, Weyertal 115b, 50931 Cologne, Germany; s.vonkarstedt@uni-koeln.de; 3 CECAD Cluster of Excellence, University of Cologne, Joseph-Stelzmann-Straße 26, 50931 Cologne, Germany; 4 Center of Molecular Medicine Cologne, Medical Faculty, University Hospital of Cologne, Weyertal 115b, 50931 Cologne, Germany

**Keywords:** S100 proteins, Ca^2+^-dependent signalling, biomarkers, cancer therapy

## Abstract

S100 proteins are widely expressed small molecular EF-hand calcium-binding proteins of vertebrates, which are involved in numerous cellular processes, such as Ca^2+^ homeostasis, proliferation, apoptosis, differentiation, and inflammation. Although the complex network of S100 signalling is by far not fully deciphered, several S100 family members could be linked to a variety of diseases, such as inflammatory disorders, neurological diseases, and also cancer. The research of the past decades revealed that S100 proteins play a crucial role in the development and progression of many cancer types, such as breast cancer, lung cancer, and melanoma. Hence, S100 family members have also been shown to be promising diagnostic markers and possible novel targets for therapy. However, the current knowledge of S100 proteins is limited and more attention to this unique group of proteins is needed. Therefore, this review article summarises S100 proteins and their relation in different cancer types, while also providing an overview of novel therapeutic strategies for targeting S100 proteins for cancer treatment.

## 1. The S100 Family

In 1965, Blake W. Moore isolated a protein from the bovine brain to identify specific proteins of the nervous system. Since it was soluble in 100% saturated ammonium sulfate at neutral pH, the protein was named “S-100” [1]. Isobe et al. later showed that this S100 protein formed a dimer, consisting of two homologous yet different molecules, defined as S100A and S100B [2,3]. Further research from the past decades revealed that their expression was not limited to nervous tissue but that S100 proteins are found in various tissues exclusively in vertebrates. Subsequently, many more S100-related proteins were discovered, and currently, 25 family members are known [4]. It has since been shown that these proteins are involved in a variety of different pathways, thereby playing a critical role in essential cellular processes, such as proliferation, apoptosis, differentiation, and inflammation [5].

### 1.1. The S100 Family Members

#### 1.1.1. Structure

S100 proteins are classified as EF-hand motif calcium-binding proteins and represent the largest subgroup within the EF-hand superfamily [6]. Typically, S100 members form homodimers, and only a few heterodimers are known, such as Moore’s S100A1/S100B protein and the S100A8/S100A9 dimer [4,7]. S100G is an exception to the rule, as it only exists as a monomer [7,8]. Van der Waals interactions stabilise the dimers, and the formation of higher-order oligomers is also observed [6,8].

Each monomer is formed by two EF-hand motifs, of which each EF-hand motif consists of a Ca^2+^-binding loop flanked by α-helices (helix–loop–helix) [9]. The N-terminal EF-hand (helix I–loop I–helix II) is S100-specific and has a lower Ca^2+^-binding affinity, due to 14 amino acid residues, while the canonical C-terminal EF-hand (helix III–loop II–helix IV) with 12 amino acid residues has a high Ca^2+^-binding affinity [9,10,11]. The two EF-hands are connected by a hinge region [9], and helix I and helix IV display the dimerisation interface [10]. When calcium binds to loop II of the C-terminal EF-hand, the orientation of helix III shifts and exposes a hydrophobic pocket, which is necessary for target binding [6,7,11]. An S100 homodimer is usually supplied with two symmetrical hydrophobic binding sites that recognise two identical target molecules [7,10,12]. S100A10 seems to be the only family member who is active in a calcium-independent manner, as it lacks a Ca^2+^-binding site and remains in an open conformation when calcium is absent [5,7,13]. Besides calcium, some S100 proteins, for example, S100A7, S100A12, and S100B are capable of binding other divalent metal ions such as zinc (Zn^2+^), copper (Cu^2+^), and manganese (Mn^2+^), which is thought to play a role in the formation of oligomers [4,14]. Moreover, the sequestering of nutrient transition metals, such as Zn^2+^ and Mn^2+^, leads to the growth inhibition of microbial pathogens [6]. In this context, Nakashige et al. demonstrated that calprotectin (CP), an S100A8/S100A9 oligomer, is also capable of binding iron (Fe^2+^) [15]. However, so far, this finding is ambiguous in comparison with previous studies [16,17]. 

Although S100 family members share high sequence similarities [10] and show similar folding behaviour, they differ regarding their shape and charge [8]. The fact that S100 proteins show a wide diversity of target proteins is to be explained by the specific target binding sites [6,12], mainly differing in the hinge region and the C-terminal extension [9], as well as the individual expression profile of each S100 family member [6].

#### 1.1.2. Expression and Regulation

So far, S100 proteins could only be found in vertebrates [6,8]. In humans, the genes for the S100A subfamily (*S100A1*–*S100A16*) are clustered on chromosome 1q21, while the genes of other S100 members are located on chromosomes 21q22 (*S100B*), Xp22 (*S100G*), 4p16 (*S100P*), and 5q13 (*S100Z*) [18,19]. Generally, an S100 gene consists of three exons and two introns, of which exon 1 is not translated, whereas exon 2 and exon 3 encode the EF-hand structures [20]. 

In contrast to Moore’s initial hypothesis that S100 proteins are only expressed in the nervous system, it was shown that S100 family members are widely expressed across various tissues. However, each S100 family member seems to have a particular expression pattern, and expression levels vary from cell type to cell type. For example, S100A9 is primarily expressed in immune cells [21], while S100A3 is mainly found in hair cuticular cells [22]. Some S100 proteins are even expressed in a cell cycle-dependent manner, for example, S100A2 is nearly absent in the G_0_ phase, but expression increases in the early G_1_ and S phase within epithelial cells [23]. 

The clustered genes of the S100A subfamily on chromosome 1q21 are part of the epidermal differentiation complex (EDC) and thereby regulated by a pool of transcription factors (e.g., Klf4, Grhl3, Arnt) [24]. Nevertheless, the cell-specific expression of these genes indicates that other factors are involved in the regulation of the *S100* genes, In this context, epigenetic features such as DNA methylation could be observed. Although these epigenetic changes have not yet been fully understood, it is presumed that inter alia S100 genes are silenced by methylation of the cytosine within CpG pairs of regulatory regions [20]. Additionally, the expression of some S100 family members seems to be regulated by microRNA (miRNA). Choe et al. showed that S100A4 is negatively controlled by miRNA-124 [25]. Recently, Wen et al. described S100B downregulation by miR-135b in cerebral palsy rat models [26]. A better understanding of the physiology of S100 proteins also demonstrated that extracellular stimuli such as growth factors and cytokines, as well as intracellular signalling cascades, can influence S100 protein levels [27,28,29]. An overview of the different expression profiles of each S100 family member is given in Table A1, which can be found in the Appendix. 

Unlike other EF-hand proteins, S100 members can not only be found inside cells but also in the extracellular space and interacting with receptors [5]. A potential active mechanism of secretion has not been fully understood since S100 proteins lack the structural sequence for the classical endoplasmic reticulum (ER)/Golgi secretory pathway, but cellular events leading to membrane ruptures such as passive cellular necrosis or regulated types of necrosis or tubulin-dependent translocation are considered in this context [30,31].

#### 1.1.3. Functions

S100 proteins interact with a high number of different targets and are thereby involved in various cellular processes, including Ca^2+^ homeostasis, proliferation, differentiation, apoptosis, inflammation, and cell migration [5]. The different functions of each S100 family member can be found in Table A1 in the Appendix. 

Ca^2+^ is involved in almost every cellular process, and elevated Ca^2+^ concentrations lead to cell death. Therefore, tight regulation of Ca^2+^ levels is essential [32]. S100 proteins secure cellular Ca^2+^ homeostasis not only by binding and transporting free intracellular Ca^2+^ to and from the plasma membrane, but also by interacting with transmembrane proteins such as the plasma membrane Ca^2+^ transport ATPase (PMCA1b), the transient receptor potential vanilloid 6 (TRPV6), and the Na^+^/Ca^2+^ exchanger (NCX1) [32,33,34,35]. A prominent example is S100A1, as it modulates the contractile performance of cardiomyocytes by interacting with the sarcoplasmic reticulum ATPase (SERCA2a) and the myocardial ryanodine receptor 2 (RyR2), which improves the systolic release and diastolic uptake of Ca^2+^ [36,37]. 

Furthermore, the S100 family is involved in inflammation and immune response. S100A8, S100A9, and S100A12 fulfil the characteristics of danger-associated molecular patterns (DAMPs), also called alarmins [38,39]. DAMPs are danger signals released by damaged, infected, or dying cells and trigger an inflammatory response [39]. The majority of extracellular S100 proteins interact with the receptor for advanced glycation end products (RAGE) and Toll-like receptor 4 (TLR4). The binding of S100A9, S100A8/S100A9, and S100A12 to these receptors stimulates nuclear factor “kappa-light-chain-enhancer” of activated B-cells (NF-kB) signalling, which results in the upregulated expression of cytokines and pro-inflammatory factors, such as interleukin-1β (IL-1β) or tumour necrosis factor α (TNFα) [29,40,41]. Further, it was shown that S100A7, S100A8, S100A9, and S100A15 have chemotactic features, attracting neutrophils and lymphocytes [23,28,40,42,43]. S100A7, S100A12, and S100A15 additionally support the initial immune response by reducing the survival of pathogens such as *Escherichia coli* (*E. coli*) [23,28]. In principle, S100B does not act chemotactically, but extracellular S100B was found to encourage microglia migration by stimulating the expression and release of chemokines [44].

The promotion of proliferation by S100 proteins is also often mediated in a RAGE-dependent manner, inducing NF-κB and mitogen-activated protein kinase (MAPK) signalling. This leads to the transcription of growth factors and survival proteins [45,46,47,48]. While extracellular S100A11 also induces RAGE-mediated transcription of the epidermal growth factor (EGF), intracellular S100A11 regulates the inhibition of proliferation [49,50]. To this end, S100A11 binds to nucleolin, which then allows the transcription of p21, resulting in the inhibition of DNA synthesis [50]. 

Additionally, it was shown that some S100 proteins, namely S100A2, S100A4, S100A14, and S100B interact with p53 [51,52,53,54]. The transcription factor p53 is an important tumour suppressor, and among other functions also regulates DNA replication and induces the transcription of pro-apoptotic proteins such as phorbol-12-myristate-13-acetate-induced protein 1 (NOXA) and p53 upregulated modulator of apoptosis (PUMA) [55]. Thereby, S100 proteins can be linked to the regulation of intrinsic apoptosis. S100A6 and S100A14 activate RAGE-mediated production of reactive oxygen species (ROS), also resulting in cell death [27,46].

### 1.2. Clinical Relevance of S100 Proteins

Although the function of some family members (e.g., S100Z) remains unknown and the complex roles of S100 proteins are not yet fully unravelled, the diversity and importance of S100 proteins are widely accepted. Consequently, any dysregulation can have severe outcomes, and S100 proteins are linked to numerous pathologic conditions and diseases. Therefore, they also show to be promising markers for diagnostic and possible novel targets for therapy, as to be discussed in the following chapter.

As previously described, S100 proteins are involved in inflammation and immune response. Therefore, it is comprehensible that S100 proteins also play a role in inflammatory disorders. Rheumatoid arthritis (RA) is the most common rheumatic disease and destroys bone and cartilage due to chronic inflammation [56]. Especially the family members S100A8 and S100A9 seem to be key molecules in the progress of RA due to their role as DAMPs. S100A8/S100A9 levels are useful biomarkers to monitor the disease, and serum concentrations can predict the response to therapeutic drugs such as methotrexate, a commonly used drug for RA treatment, and adalimumab, a TNFα-blocking antibody [56,57,58]. The testing of S1008/S100A9 in stool samples is an established method to diagnose inflammatory bowel diseases (IBDs), a chronic inflammatory disease of the gut. Although it is not possible to discriminate between different types of bowel disease based on S100A8/S100A9 testing, this non-invasive method is very accurate and favoured in the pediatric field [59,60]. Additionally, S100 family members could also be linked to allergies, cystic fibrosis, psoriasis, and several other inflammatory diseases [61]. 

Pathologic S100 concentrations can also be detected in neurological diseases [61]. The Scandinavian Neurotrauma Committee (SNC) recommends using S100B as a biomarker in addition to the classical anamnesis to identify patients with a high risk for intracranial injuries (ICI) after a mild traumatic brain injury (TBI) [62]. In this context, serum S100B demonstrates high sensitivity for the detection of ICI after mild TBI and analysing S100B levels brings great benefits, since only 8% of patients with mild TBI suffer from ICIs. Therefore, the application of cost-intensive computerised tomography (CT) can be reduced, and patients are protected from unnecessary exposure to radiation [63,64]. Changes in S100B levels were also found in patients with psychiatric diseases, such as schizophrenia, depression, and bipolar disorder, but more studies are needed to establish S100B as a reliable clinical biomarker [65]. Evaluated levels of several S100 proteins could also be observed in the brain of Alzheimer’s patients. These S100 family members, namely S100A1, S100A6, S100A7, S100A8, S100A9, S100A12, and S100B, seem to be involved in the progression of Alzheimer’s disease (AD), including the formation of amyloid aggregates, and could thereby be promising regarding new therapeutic approaches [66]. 

S100A1 seems to be an essential factor concerning cardiological diseases since it is involved in the contractile performance of cardiomyocytes [36,37]. Fan et al. demonstrated that patients showing ST-segment elevation myocardial infarction (STEMI) in the electrocardiogram (ECG) also had increased levels of S100A1 and that this S100 family member could complement current biomarkers [67]. It was also shown that S100A1 concentrations rise during early-stage acute myocardial ischemia, followed by a quick decrease, and thereby S100A1 could represent an early biomarker [68]. In addition, studies with S100A1 gene therapy in animal models predict a promising new strategy for the treatment of heart failure [69]. 

Furthermore, changes in S100 expression could also be observed in several cancer types, and S100 proteins seem to play a crucial role in the development of tumours. This context will be discussed in detail in the following section.

## 2. S100 Proteins in Cancer

In the 1980s, soon after the identification of the first S100 family members, the presence of S100 proteins was also observed in different cancer types, drawing rising attention to this group of calcium-binding proteins within the oncological field. In this context, S100 expression was, for example, detected in metastatic melanoma [70,71], renal cell carcinoma [72], and breast cancer [73]. Moreover, an S100 protein expressed in mouse Ehrlich ascites-tumour cells was identified as S100A6 [74], and S100P was found in the cell nuclei of brain tumours [75]. Further research from the past decades demonstrated that dysregulation of S100 proteins, typically upregulation, can be connected to tumour growth, angiogenesis, and metastasis [76]. Several S100 proteins could also be linked to drug resistance and seem to be involved in mediating the response to chemotherapy [77]. Each cancer type shows a specific S100 expression profile, and the different S100 family members function individually in a tissue-dependent manner. S100A2, for example, operates as a tumour suppressor in oral cancer while it promotes tumour growth in lung cancer [78]. The role of S100 proteins has been widely examined in breast cancer, lung cancer, and melanoma, but pathologic S100 signalling could also be observed in additional cancer types, such as ovarian, colorectal, and pancreatic cancer.

### 2.1. S100 Proteins in Breast Cancer

Breast cancer is the most common cancer type among women [79], and especially metastatic breast cancers are highly lethal [80]. It was shown that several S100 family members, including S100A2, S100A4, S100A6, S100A7, S100A8, S100A9, and S100A11 are up- or downregulated in breast cancer compared with healthy tissue, suggesting that S100 proteins play a crucial role in tumour development and progression [81,82] and as predictive biomarkers [83,84,85,86,87,88].

Zhang et al. analysed S100 mRNA expression in breast cancer patients (*n* = 1117), using the online databank Gene Expression Omnibus. Intriguingly, the expression of S100 proteins correlates with the overall survival (OS) of patients, depending on cancer subtype and clinicopathologic features. For example, high mRNA levels of S100A8 and S100A9 predict worse prognosis in the luminal A-type breast cancer, while increased levels of S100A10, S100P, and S100Z showed shorter OS in patients with the basal-like subtype. In contrast, longer OS was observed for patients with luminal a subtype expressing high levels of S100A1, S100A2, and S100A6 and for patients with the basal-like subtype expressing high levels of S100A14. Overall, S100A1 and S100A6 seem to predict better prognosis and S100A8, S100A9, S100A11, and S100P seem to worsen OS rates [84]. Zhong et al. found a correlation between S100A8 and lymph node metastasis in breast cancer and therefore, tumour progression [85]. Further publications covering this connection identified S100B as a serum marker in endocrine-resistant breast cancer [86] and elevated S100B serum levels as a negative prognostic value for breast cancer [87,88]. Nevertheless, a collective expression pattern of all S100 members together might have higher prognostic value than the single proteins [82]. 

S100A4 is highly involved in metastasis and invasion of many different cancer types, including breast cancer [89,90]. Barraclough et al. early showed that S100A4 is associated with a metastatic phenotype using a rat mammary model. It was suggested that the underlying mechanism includes the interaction of S100A4 with cytoskeletal proteins, such as actin and myosin, thereby promoting cell motility [91]. These findings are supported by further studies, showing that intracellular S100A4 interacts with actin, non-muscle myosin heavy chain IIA (NMIIA), and tropomyosin, leading to cell migration [92,93]. Although S100A1 does not seem to be directly involved in breast cancer signalling, it was shown that S100A1 reduces the activity of intracellular S100A4 [94]. The interaction between S100A4 and matrix metalloproteinase 2 (MMP2) induces epithelial–mesenchymal transition (EMT), which is considered to be an initial step during invasion and metastasis [95]. S100A4 can not only be found in cancer cells but is also highly expressed in stromal cells of the tumour microenvironment (TME), such as fibroblasts, T-cells, macrophages, and neutrophils [81,96]. Extracellular S100A4 in the TME induces the release of pro-inflammatory factors (e.g., interleukin 6 (IL-6), interleukin 8 (IL-8), and C-X-C motif chemokine 10 (CXCL10)), which then converts monocytes into tumour-associated macrophages (TAMs), resulting in metastasis and drug resistance [97]. 

Only a few studies have been performed to investigate the signalling of S100A6 in breast cancer. However, S100A6 induces the activity of calcyclin-binding protein/Siah-1-interacting protein (Cacy/SIP), which is involved in tumour invasion and metastasis in breast cancer most likely by increasing β-catenin levels [98,99]. Another exciting approach showed that S100A6 levels decrease in human breast cancer cell line MDA-MB-231 when treated with anti-tumour agents, namely tumour necrosis factor-related apoptosis-inducing ligand (TRAIL) and etoposide, indicating that S100A6 might be a potential biomarker for apoptosis during therapy [100]. 

S100A7 was also identified as an essential protein in breast cancer cells, especially in estrogen receptor α (ERα) negative cells, where the binding to RAGE leads to activation of extracellular signal-regulated kinase (ERK) and NF-κB signalling, resulting in tumour growth and enhanced metastasis [101]. Moreover, increased NF-κB activity was observed in S100A7-overexpressing breast cancer cells, associated with evaluated levels of NF-κB target genes matrix metalloproteinase 9 (MMP9) and vascular endothelial growth factor (VEGF), resulting in proliferation and invasion [102]. Further, the binding of S100A7 and RAGE leads to the recruitment of TAMs, which then promote further tumour growth, angiogenesis, and metastasis by expressing chemokine (C-C motif) ligand 2 (CCL2), cyclooxygenase-2 (COX2), and VEGF [101,103]. Intracellular S100A7 interacts with the transcriptional cofactor constitutive photomorphogenesis 9 (COP9) constitutive photomorphogenic homolog subunit 5 (COPS5), also known as c-Jun activation domain-binding protein-1 (Jab1), which then accumulates in the nucleus and promotes the expression of AP-1 and NF-κB, resulting in enhanced tumour growth and invasion [104].

**Figure 1:**
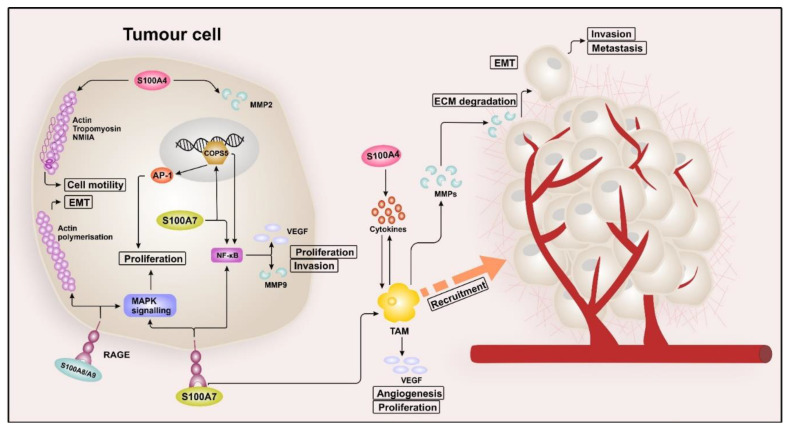
S100 signalling in breast cancer. Intracellular S100A4 interacts with cytoskeletal proteins, such as actin, non-muscle myosin heavy chain IIA (NMIIA), and tropomyosin, which promotes cell motility. Besides, S100A4 can induce epithelial–mesenchymal transition (EMT) by regulating the expression of matrix metallopeptidases 2 (MMP2), leading to invasion and metastasis. Extracellular S100A4, located in the tumour microenvironment (TME), induces the release of pro-inflammatory factors (e.g., IL-6, IL-8, and CXCL10). These cytokines then convert monocytes into tumour-associated macrophages (TAMs), which in return, promote EMT, proliferation, and drug resistance of the tumour cells. The binding of extracellular S100A7 to the receptor for advanced glycation end products (RAGE) induces mitogen-activated protein kinase (MAPK) and nuclear factor “kappa-light-chain-enhancer” of activated B-cells (NF-κB) signalling, resulting in tumour growth and metastasis. Increased NF-κB activity was observed in S100A7-overexpressing breast cancer cells, associated with evaluated levels of matrix metalloproteinase 9 (MMP9) and vascular endothelial growth factor (VEGF), resulting in proliferation and invasion. The binding of S100A7 and RAGE also leads to the recruitment of TAMs, which then promote further tumour growth, angiogenesis, and metastasis by expressing chemokine (C-C motif) ligand 2 (CCL2), cyclooxygenase-2 (COX2), and VEGF. Intracellular S100A7 interacts with the transcriptional cofactor COP9 constitutive photomorphogenic homolog subunit 5 (COPS5), which in turn promotes the expression of AP-1 and NF-κB, resulting in enhanced tumour growth and invasion. S100A8/S100A9 enhances breast cancer cell growth by inducing MAPK signalling in a RAGE-dependent manner. In addition, RAGE mediates cell migration by promoting actin polymerisation and EMT, leading to metastasis and invasion.

The binding of S100A8/S100A9 to RAGE promotes breast cancer cell growth by inducing MAPK signalling. In addition, RAGE then mediates cell migration by promoting actin polymerisation and EMT, resulting in metastasis and invasion [105]. Although not all S100 proteins have been studied intensively in this context, the current knowledge of S100 signalling in breast cancer is summarised in Figure 1.

### 2.2. S100 Proteins in Lung Cancer

**Figure 2:**
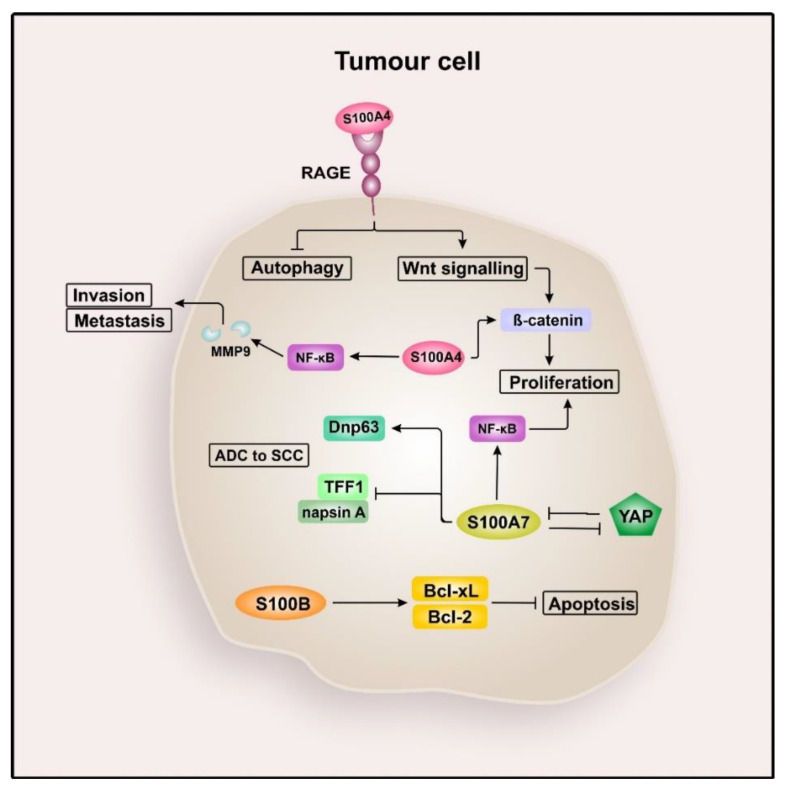
S100 signalling in lung cancer. Extracellular S100A4 inhibits autophagy and induces Wnt signalling by interacting with the receptor for advanced glycation end products (RAGE) and intracellular S100A4 additionally activates β-catenin, resulting in increased proliferation and enhanced viability of lung cancer cells. S100A4 also induces the expression of (MMP9) by activating nuclear factor “kappa-light-chain-enhancer” of activated B-cells (NF-κB), thereby promoting invasion and metastasis. S100A7 is most likely involved in adenocarcinoma (ADC) to squamous cell carcinoma (SSC) transdifferentiation of lung cancer cells, by upregulating the SSC marker DNp63 and downregulation of the ADC markers thyroid transcription factor 1 (TTF1) and aspartic proteinase napsin (napsin A). In this context, an inverse correlation of S100A7 and yes-associated protein (YAP) was observed. Moreover, S100A7 seems to activate NF-κB-dependent cell proliferation. Within lung cancer-derived brain metastasis cells, S100B was shown to upregulate the expression of B-cell lymphoma 2 (Bcl-2) and B-cell lymphoma extra-large (Bcl-xL), indicating that S100B is capable of suppressing apoptosis.

Lung cancer is the most common cancer type with a high mortality rate [79], mostly due to lacking diagnostic means for efficient early detection [106]. There are two major groups of lung cancer: non-small cell lung cancer (NSCLC), which is diagnosed in about 80% of all lung cancer patients, and small cell lung cancer (SCLC) [107,108]. It was shown that S100A1, S100A2, S100A3, S100A4, S100A6, S100A7, S100A8/S100A9, S100A10, S100A11, S100B, and S100G are overexpressed in NSCLC, and except S100B, all are predictive for poor survival [109]. The function of several S100 proteins in lung cancer remains unclear. However, some family members have been studied in this context and are summarised in Figure 2.

The studies on S100A2 in NSCLC presented conflicting data [110]. For example, Feng et al. showed that S100A2 was downregulated in NSCLC cell lines and detected CpG methylation in the promoter region of the S100A2 gene, indicating that S100A2 was suppressed during early-stage carcinogenesis [111]. In contrast, Heighway et al. demonstrated that S100A2 was strongly expressed in primary NSCLC tissue [112], and Bulk et al. later showed that S100A2 acts as a metastasis inducer in mouse models [113]. In 2014, Hountis et al. introduced a dual role concept for S100A2 in lung cancer, implicating that S100A2 is primarily expressed in the nucleus at an early stage of NSCLC, where it mediates resistance to p53-dependent apoptosis and inhibits tumour-promoting genes (e.g., PA1-1 and vimentin) and in later stages relocates to the cytoplasm in a Ca^2+^-dependent manner [114]. 

S100A4 seems to be an important player in the development and metastasis of lung cancer, promoting tumour cell proliferation and motility [115,116]. The overexpression of S100A4 in lung adenocarcinoma (ADC), a subtype of NSCLC, was linked to reduced OS of these patients [117]. In vitro studies revealed that extracellular S100A4 inhibited autophagy and induced Wnt signalling in a RAGE-dependent manner and intracellular S100A4 additionally activated β-catenin, resulting in increased proliferation and enhanced viability of lung cancer cells [118]. Further, in vitro studies showed that the depletion of S100A4 resulted in decreased NF-κB signalling and inhibition of matrix metalloproteinases 9 (MMP9), demonstrating that the S100A4/NF-κB/MMP9 signalling axis promotes invasion [117]. 

Regarding the role of S100A7 in lung cancer, it was shown that this protein is highly expressed in lung squamous cell carcinoma (SCC), an NSCLC subtype, and that knockdown of S100A7 resulted in decreased NF-κB-dependent cell proliferation [119]. In vitro, Wang et al. demonstrated that S100A7 expression was induced by the Hippo pathway and depletion of S100A7 lead to suppression of DNp63, a marker for SSC, while the ADC markers TFF1 and napsin A were upregulated, and an inverse correlation of S100A7 and yes-associated protein (YAP) was observed. In summary, these data implicate that S100A7 is an essential player in cellular plasticity and ADC to SSC transdifferentiation [120].

S100B was introduced as a possible biomarker for brain metastasis in lung cancer patients [121,122]. Further investigation of S100B in an ADC-derived brain metastasis cell line showed that depletion of S100B resulted in the downregulation of pro-survival factors, namely B-cell lymphoma 2 (Bcl-2) and B-cell lymphoma extra-large (Bcl-xL), both inhibitors of apoptosis, indicating that S100B suppresses apoptosis in these cancer cells. In this study, it was moreover observed that S100B correlates with enhanced proliferation, migration, and invasion [123]. In contrast, Liu et al. showed that increased mRNA levels of S100B predicted better OS in patients with NSCLC [109]. Therefore, the molecular mechanisms of S100B in lung cancer need to be further evaluated to classify this protein in a clinical context.

### 2.3. S100 Proteins in Malignant Melanoma

Malignant melanoma arises from mutated melanocytes, and although melanoma is exceedingly rare (<2% of all skin malignant tumours), it is the deadliest form of skin cancer [124,125]. The expression of several S100 family members, namely S100A1, S100A2, S100A4, S100A6, S100A7, S100A8, S100A9, S100A10, S100A11, S100A13, and S100B, were detected in melanoma tissue, partially depending on the cancer stage [76,126]. 

As previously discussed, S100A4 is involved in the metastatic processes in other cancer types, and similar observations were made in melanoma. Upregulation of S100A4 correlates with the upregulation of RAGE in metastatic melanoma cells in vitro, and elevated levels of S100A4 and RAGE are associated with high tumour burden in vivo [127,128]. It was shown that extracellular S100A4 activates NF-κB in a RAGE-dependent manner, resulting in the release of TNFα [129]. S100A4 also induces the secretion of further paracrine factors, such as IL-8 and CCL2, which in return, promote angiogenesis and recruitment of monocytes, creating an inflammatory milieu in the tumour microenvironment [130]. Moreover, it was revealed that extracellular S100A4 decreases the expression of occludin and VE-cadherin in endothelial cells (ECs), thereby disrupting cell–cell adhesion. This enables melanoma cells to transmigrate through the EC monolayer into the bloodstream [128]. In summary, S100A4 induces metastatic signalling and promotes cell migration in malignant melanoma. 

When monitoring stage IV melanoma patients during immunotherapy with ipilimumab, the heterodimer S100A8/S100A9 attracted attention, as high serum levels of it in early stages of treatment predicted worse response [131]. The release of S100A8/S100A9 can be induced by UV radiation- exposed keratinocytes, and extracellular S100A8/S100A9 then promotes proliferation and migration of melanocytes via RAGE-dependent signalling [132]. It could be demonstrated that interaction between S100A8/S100A9 and RAGE leads to increased levels of the metalloproteinases MMP2, MMP9, and MMP14 in melanoma cells, which enhances metastatic properties [133]. Besides RAGE, novel ligands for S100A8/S100A9 were found. S100A8/S100A9 binds to the melanoma cell adhesion molecule (MCAM), a highly expressed cell adhesion molecule in melanoma [134]. This interaction activates mitogen-activated protein kinase kinase kinase 8 (MAP3K8), also termed tumour progression locus 2 (TPL2), which conversely stimulates the transcription factor ETS translocation variant 4 (ETV4), leading to induction of MMP25 and promoting melanoma lung metastasis [135]. The homodimer of S100A9 additionally interacts with extracellular matrix metalloprotease inducer (EMMPRIN), which activates TNF receptor-associated factor (TRAF2)-dependent NF-κB signalling and the upregulation of cytokines such as TNFα, CXCL1, CXCL2, and CXCL3, resulting in metastasis [136]. MCAM and EMMPRIN are both highly expressed in melanoma and inhibition of the interaction between these receptors and S100A8/A9 could suppress lung metastasis in vivo, thereby representing an interesting target for therapeutic approaches [137]. 

**Figure 3:**
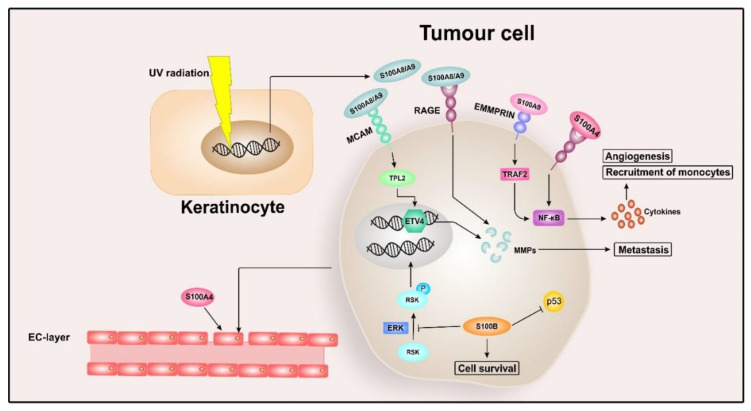
S100 signalling in melanoma. Extracellular S100A4 binds to the receptor for advanced glycation end products (RAGE) and thereby activates nuclear factor “kappa-light-chain-enhancer” of activated B-cells (NF-κB), resulting in the release of cytokines such as tumour necrosis factor α (TNFα). These cytokines then promote angiogenesis and recruitment of monocytes, creating an inflammatory milieu in the tumour environment. Furthermore, extracellular S100A4 also decreases the expression of occluding and vascular endothelial cadherin (VE)-cadherin in endothelial cells (ECs), thereby disrupting cell–cell adhesion and enabling the tumour cells to transmigrate through the EC monolayer into the bloodstream. The release of S100A8/S100A9 can be induced by UV radiation-exposed keratinocytes, and extracellular S100A8/S100A9 then promotes proliferation and migration of melanocytes via RAGE signalling. The interaction between S100A8/S100A9 and RAGE can lead to increased levels of the metalloproteinases MMP2, MMP9, and MMP14 in melanoma cells, thereby enhancing metastatic properties. In addition to RAGE, S100A8/A9 also binds to the melanoma cell adhesion molecule (MCAM), thereby activating tumour progression locus 2 (TPL2) and stimulating the transcription factor ETS translocation variant 4 (ETV4), leading to the induction of MMP25 and promoting melanoma lung metastasis. The homodimer of S100A9 additionally interacts with extracellular matrix metalloprotease inducer (EMMPRIN), which activates TNF receptor-associated factor (TRAF2)-dependent NF-κB signalling and the upregulation of cytokines such as TNFα, chemokine (C-X-C motif) ligand 1 (CXCL1), CXCL2, and CXCL3, resulting in metastasis. S100B inhibits the phosphorylation of ribosomal S6 kinase (RSK) by extracellular signal-regulated kinase (ERK) in a Ca^2+^-dependent manner so that RSK remains in the cytoplasm, leading to improved tumour survival. S100B also inhibits p53 activity at the protein level, thereby preventing p53-dependent apoptosis.

S100B is described to be a prognostic marker for the advanced stages of melanoma, especially for distant metastasis, and increased serum S100B levels are associated with shorter disease-free survival (DFS) and OS [138,139,140]. Investigating the molecular mechanisms of S100B in melanoma cells, it could be revealed that S100B interacts with ribosomal S6 kinase (RSK). RSK is a downstream target of ERK, and S100B inhibits phosphorylation of RSK by ERK in a Ca^2+^-dependent manner so that RSK remains in the cytoplasm and does not relocate to the nucleus. This results in increased activity of RSK in the cytoplasm, leading to improved tumour survival [141]. In vitro studies also revealed that increased levels of p53 in malignant melanoma cells promote expression of S100B, and within a negative feedback loop, S100B inhibits p53 activity at the protein level, thereby preventing p53-dependent apoptosis [54,142]. While p53 is mutated in most cancer types, 80% of melanomas express wild-type p53 [143], and therefore S100B might be an attractive target for new therapeutic approaches in this context. An overview of S100 signalling in malignant melanoma is given in Figure 3. 

### 2.4. S100 Proteins in Ovarian Cancer

Recent studies investigating mRNA expression of S100 proteins in correlation with the survival of ovarian cancer patients demonstrated that high levels of the S100 family members S100A2, S100A10, S100A11, S100A15, S100A16, and S100B predict worse OS, while S100A1, S100A3, S100A5, S100A6, S100A13, S100G, and S100Z are associated with longer OS, partially depending on cancer subtype and clinicopathological features [144,145]. However, the underlying mechanisms of these S100 proteins in the context of ovarian cancer have not yet been fully understood. 

Nevertheless, among others, S100A3, S100A10, and S100B were identified to be related to drug resistance in ovarian cancer [146,147,148]. Elevated expression of S100A3 was found primarily in ovarian cancer cells resistant to cisplatin and paclitaxel, both standard chemotherapeutic drugs, as well as topotecan, a chemotherapeutic agent commonly used in second-line treatment [146]. S100A10 was 1 of 11 genes associated with multidrug resistance in ovarian primary serous carcinoma tissue, although further studies are needed to validate these data, as this relation could not be confirmed in a group with a smaller sample size [147]. In vitro studies could demonstrate that S100B contributes to cisplatin resistance in ovarian cancer stem cells (OCSC) by inhibiting p53 and therefore promoting multidrug resistance gene 1 (MDR1) and MDR-associated protein 1 (MRP1) [148,149].

Moreover, it was shown that ovarian cancer patients with high expression of cytoplasmic S100A10 and stromal Annexin A2 have a 3.4-fold increased risk of progression and a 7.9-fold higher risk of cancer-related death [150]. Annexin A2 and S100A10 form a heterotetramer referred to as AIIt, which interacts with the tissue plasminogen activator (tPA). This leads to the production of plasmin, which results in ECM degradation, EMT, and angiogenesis [151,152]. In addition, S100A10 is able to promote MMPs, altogether provoking cell migration and invasion [153].

### 2.5. S100 Proteins in Colorectal Cancer

Mounting evidence suggests that S100 proteins play a crucial role in colorectal carcinogenesis, through promoting proliferation, migration, and invasion [154]. Several S100 family members seem to be promising biomarkers, for example, high expression of S100A4 is associated with poor survival and increased aggressiveness when studying colorectal carcinoma (CRC) patients [155,156]. S100A8/A9 could be a reliable marker for diagnosis, and postoperative monitoring of CRC [157] and S100B can predict early relapse in stages II and III postoperative colon cancer patients [158].

Regarding the role of S100A4 in colorectal cancer, it comes as no surprise that this protein seems to be related to metastasis, as similar observations were made in other cancer types, such as breast and lung cancer [90,115,159]. It was shown that extracellular S100A4 promotes ERK and hypoxia signalling in a RAGE-dependent manner, mediating colon cancer cell motility [160]. Further, S100A4 is capable of activating the phosphoinositide 3-kinase/Akt/mechanistic target of the rapamycin pathway (PI3K/Akt/mTOR), leading to increased levels of VEGF and decreased E-cadherin, which triggers tumour progression and cell migration [161]. 

Furthermore, high protein expressions of S100A8 and S100A9 were found to be associated with differentiation and lymph node metastasis in CRC tissue. This overexpression correlates with increased levels of β-catenin [162]. Additionally, S100A8/A9 interaction with RAGE activates NF-κB and MAPK signalling, leading to upregulation of chemokines, such as CXCL1, CLC5, and CLC7, resulting in the formation of a premetastatic niche and tumour progression [163]. 

Recent studies also unravelled the involvement of S100P in tumorigenesis, metastasis, and invasion of CRC [164]. In this context, thioredoxin-1 (Trx-1) and β-tubulin were introduced as novel downstream targets of S100P, and interaction between these proteins led to improved cell viability and promotion of cell migration [165,166].

### 2.6. S100 Proteins in Pancreatic Cancer

S100 proteins were shown to be involved in tumour progression and metastasis of pancreatic cancer [164,167]. S100A2, for example, is overexpressed in pancreatic cancer tissue and seems to be a good predictor regarding the response to pancreatectomy of pancreatic cancer patients [168,169]. S100A6 was found to be overexpressed, especially in the early phase of carcinogenesis, and high nuclear S100A6 levels predict poor survival of pancreatic cancer patients [170,171]. Similar observations were made for S100A11, as the expression is detected in the earlier stages and seems to decrease during tumour progression [172]. Besides, it has been suggested that the expression of S100A11 correlates with poor prognosis of pancreatic cancer patients after surgical resection [173]. Furthermore, evidence indicated that S100P could be a helpful marker for the diagnosis of pancreatic cancer, as it is specifically expressed in cancer cells [174,175].

Several studies also introduced S100A4 as a potential biomarker, as its expression correlates with malignancy, metastasis, invasion, and overall poor prognosis in pancreatic cancer [176,177,178]. Furthermore, it was found that S100A4 is overexpressed in pancreatic adenocarcinoma, most likely due to hypomethylation at intron 1 of the S100A4 gene [179]. S100A4 promoted cell growth and cell motility and inhibited apoptosis in vitro [180,181]. The knock-down of S100A4 did not only induce apoptosis but also increased gemcitabine sensitivity [182]. By studying the underlying mechanisms of S100A4 in pancreatic cancer, it could be shown that S100A4 is upregulated by sonic hedgehog (Shh)-Gli-1 signalling as part of the hedgehog pathway, eventually resulting in EMT and further leading to metastasis [183]. Recent in vivo studies, including an orthotopic human pancreatic cancer xenograft mouse model, revealed that S100A4 activates focal adhesion kinase (FAK) and Src kinase, both involved in tumour progression and metastasis [184]. 

High expression of S100A8 and S100A9 was found in the stroma cells, mainly monocytes, of pancreatic cancer, and the number of S100A8 positive stroma cells correlated with the Smad4 status of the tumour. Smad4 is a key mediator for TGFβ, a pro-apoptotic protein involved in pancreatic tumour progression, and tumours without Smad4 expression showed a decreased number of S100A8+ stroma cells [185,186]. However, further studies are needed to fully understand the underlying mechanisms.

### 2.7. S100 Proteins in Other Cancer Types

A link between S100 proteins and additional cancer types, such as prostate cancer, bladder cancer, liver cancer, and oral cancer, could be made, although these relations have not yet been examined as closely as in previously described cancer types. Nevertheless, the current knowledge of S100 family members in these cancer diseases will be summarised in the following chapter. 

In prostate cancer, S100A8 and S100A9 are upregulated and might be helpful markers in the early stage of prostate tumour progression [187]. In vitro studies showed that the heterodimer S100A8/A9 is secreted by prostate cancer cells, and extracellular S100A8/A9 activates NF-κB and MAPK signalling [188]. In contrast, S100A6 is present in prostate basal cells but absent in malignant prostate cells, indicating that S100A6 could expand the current spectrum of biomarkers for diagnosis [189]. However, more studies are needed to validate these findings and to further understand the role of S100 proteins in prostate cancer. 

PCR-based analysis of human bladder cancer tissue showed that mRNA levels of S100A2, S100A3, S100A5, S1007, S100A8, S100A9, S100A14, S100A15, S100A16, and S100P were elevated, while S100A1, S100A4, and S100B were downregulated compared with normal bladder urothelium [190]. However, it is important to notice that only small sample size (*n* = 10) was examined and that a repeat of this study with a greater sample size could improve statistical stability. Nevertheless, the expression of S100A8 in bladder cancer was associated with disease progression in non-muscle-invasive bladder cancer (NMIBC) in more recent studies [191,192]. Investigating S100 proteins in bladder cancer also revealed a correlation between S100P and drug resistance, as decreased S100P levels were found in cisplatin-resistant bladder cancer cells, whereas overexpression of S100P increased sensitivity to cisplatin [193]. 

The role of S100 proteins in hepatocellular carcinoma (HCC), the most common form of primary liver cancer [194], is poorly understood so far. However, it was shown that high expression of S100A4 in association with high expression of vimentin and low expression of E-cadherin correlates with decreased OS [195]. Moreover, in vivo mouse model studies could demonstrate that the depletion of S100A4+ stroma cells, including mainly fibroblasts and macrophages, within the TME of HCC, reduced stemness of the tumour, and inflammation [196]. However, Li et al. presented evidence showing that S100A4 in cooperation with collagen I is involved in the development of fibrosis-associated HCC by upregulating stem cell marker genes, for example, Oct-4, Nanog, and SOX2, via RAGE-dependent β-catenin signalling [197]. Recently, S100A14 was introduced as a promising marker, as it was overexpressed in HCC tissue compared with healthy liver tissue and cirrhosis tissue, and therefore might be a sensitive assistant for diagnosis of HCC [198,199].

The expression of S100 proteins in oral squamous cell cancer (OSCC) remains controversial, as expression profiles vary from study to study, and further investigation is needed to identify reliable biomarkers among the S100 family members in this context [200]. Nevertheless, S100A14 seems to have tumour-suppressing properties in oral cancer, as overexpression of S100A14 in OSCC cell lines resulted in suppression of invasion, associated with the downregulation of MMP1 and MMP9, as well as decreased proliferation, correlating with upregulation of p21 and G_1_ cell cycle arrest [201,202]. 

The evaluated expression of S100 proteins could also be detected in malignant mesothelioma [203]. In this context, S100A4 was shown to be a promising biomarker, as it correlated with tumour progression, morphologic changes related to EMT, invasion, and metastasis [204]. However, more research is needed to validate the role of S100 family members in malignant mesothelioma.

Soluble factors originating from glioma cells have been linked to affecting the tolerance of microglia, which function as mediators of innate and adaptive immune responses. The activation of immunosuppressive signalling pathways such as STAT3 blocks the effector function of microglia [205,206,207,208]. There is growing evidence that the RAGE ligand S100B is another factor that plays a role in glioma progression. Low levels of S100B expressed by gliomas triggered STAT3 and blocked microglia and also macrophage activation [209]. S100B may also encourage glioma growth by TAM chemoattraction and therefore, infiltration into gliomas through the upregulation of CCL2 [210]. In a high-throughput screening cell-based S100B promoter-driven luciferase reporter assay, duloxetine was identified to inhibit S100B and CCL2 production in a mouse glioma model. The inhibitor had the potential to modulate the immune-suppressive behaviour of TAMs [211]. Moreover, it has been revealed that high S100B levels correlate with poor prognosis in recurrent glioma patients [212].

## 3. Targeting S100 Proteins for Cancer Therapy

As it is now well established that S100 proteins are widely involved in carcinogenesis, invasion, and metastasis, a broad spectrum of novel therapeutic opportunities arises. There are several different strategies for targeting the S100 family members in cancer. While some inhibitors seem to be effective by inhibiting the transcription of S100 genes, others inhibit S100 protein activity by disturbing the interaction between S100 proteins and their targets. In addition, targeting covalent modifications, such as S-nitrosylation, S–glutathionylation, and phosphorylation, could be a promising strategy, as these modifications influence the function of S100 proteins [213]. In this review, S100 inhibitors are classified into small molecule inhibitors, neutralising antibodies, and microRNA (miRNA) mimics, and a summary is given in Table 1.

### 3.1. Small Molecule Inhibitors

Small molecule inhibitors are compounds (<500 Da) able to penetrate tissue more effectively than macromolecules and are usually suitable for oral administration [245]. Although several small molecule inhibitors have been approved for cancer treatment, it is essential to further identify new compounds, to overcome drug resistance [246]. 

In this context, small molecule inhibitors were found which inhibit the transcription of S100 genes. The expression of S100A4, for example, is mediated by Wnt/β-catenin signalling, a pathway which is highly involved in many cancer types, especially colon cancer. Therefore, inhibitors were introduced to inhibit the transcription of the *S100A4* gene by interfering with the Wnt pathway [45]. Calcimycin, a calcium ionophore, and sulindac sulfide (sulindac), a nonsteroidal anti-inflammatory drug, inhibit the expression of β-catenin, leading to reduced levels of target genes, including S100A4. Treatment of mice with these inhibitors resulted in decreased tumour growth, reduced invasion, and fewer metastases of colon cancer at least partially due to lower levels of S100A4 [214,215]. Furthermore, the U.S. Food and Drug Administration (FDA)-approved anthelmintic drug niclosamide was identified to inhibit S100A4-induced metastasis formation of colon cancer, by preventing β-catenin/T-cell factor (TCF) complex formation and therefore transcription of S100A4 [216]. Niclosamide tablets are currently being studied in a phase II clinical trial for the treatment of metastasised CRC [217]. Although these inhibitors seem to be very effective in pre-clinical studies, it is vital to notice that they are not selective inhibitors of S100A4, for example, the inhibition of β-catenin also results in downregulation of cyclin D1 and the proto-oncogene c-Myc [214], which might cause unfavourable side effects [247]. 

Other small molecule inhibitors, therefore, follow the strategy of inhibiting the target interaction of S100 proteins. Trifluoperazine (TFP), a phenothiazine, for example, was introduced as an S100A4 inhibitor, as TFP and S100A4 form heterodimers and five dimers then arrange to a pentameric ring. This sequestering mechanism inhibits the interaction between S100A4 and myosin-IIA, indicating that TFP might influence cell motility [218,219]. Structure-based virtual inhibitor screening could also identify substituted 1,2,4-triazoles as potential S100A10 inhibitors, as it was demonstrated that 1,2,4-triazole competes with annexin A2 over the binding to S100A10 [224,225]. However, the effects of TFP and 1,2,4-triazoles in vitro and in vivo have yet to be demonstrated. 

Furthermore, tasquinimod, a quinoline-3-carboxamide derivative, could be identified as an S100A9 inhibitor. Tasquinimod binds to S100A9 and therefore blocks the interaction with RAGE and TLR4, which influences the TME by modulating myeloid cell populations [220,221,222]. Recently, a phase III clinical trial was completed, investigating the effect of tasquinimod on patients with metastatic castration-resistant prostate cancer (mCRPC). This study showed that treatment with tasquinimod resulted in significantly longer radiologic progression-free survival compared with the placebo group (7.0 months vs. 4.4 months), but no influence on the OS was observed (24 months for placebo vs. 21.3 months for tasquinimod treatment) [223]. 

As previously described, the interaction of S100B and p53 is a promising target, especially in melanoma [54,142,143]. In this context, pentamidine, an antiprotozoal agent, was identified to bind to the p53 binding site of S100B, thereby inhibiting S100B/p53 interaction, allowing p53 to restore tumour-suppressing properties [226,227]. Pentamidine is currently being evaluated within a phase II clinical study for the treatment of refractory wild-type p53 melanoma [228]. 

Another noteworthy S100 inhibitor is the anti-allergy drug cromolyn. Cromolyn binds to S100P, thereby inhibiting interaction with RAGE, leading to decreased proliferation, invasion, and NF-κB activity in vitro and reduced tumour growth in vivo. Moreover, cromolyn increased gemcitabine sensitivity for pancreatic cancer models [229,230]. However, high concentrations of cromolyn were needed to achieve these effects. A more recent study showed that the analogue 5-methyl cromolyn is more efficient than cromolyn, and lower doses could induce similar reactions [231].

### 3.2. Neutralising Antibodies

Therapeutic antibodies have brought great benefit to cancer therapy, as they are more selective compared with conventional chemotherapeutics, and evolving technology has enabled the development of 30 FDA-approved monoclonal antibodies for cancer treatment so far [248,249]. The anti-cancer effect of these antibodies is either achieved by immune-mediating mechanisms or by neutralising important players of tumorigenic pathways [250,251]. The latter strategy seems to be an exciting approach to target S100 proteins. 

As it was shown that S100A4 is highly involved in metastatic processes through influencing the TME, it is a favourable strategy to neutralise S100A4 activity. In this context, an anti-S100A4 antibody, named 6B12, showed immunomodulatory activity by binding S100A4 and thereby preventing T-cell attraction to the tumour side, which reduced metastasis in lung and breast cancer within experimental mouse models [232,233]. The anti-S100A4 antibody 5C3 could show similar effects as it terminated endothelial tumour growth, cell migration and angiogenesis in vitro and in vivo for pancreatic cancer and melanoma models. In addition, a synergistic effect between 5C3 and gemcitabine was observed [234]. 

Furthermore, a monoclonal antibody targeting extracellular S100A7 was designed. It could be demonstrated that this anti-S100A7 antibody, named 6F5, blocks S100A7/RAGE interaction, thereby inhibiting S100A7-mediated MMP9 activity, leading to decreased tumour growth, cell migration, and angiogenesis in a xenograft cancer model [235]. In terms of neutralising the DAMP molecules S100A8 and S100A9, monoclonal Ab45 was identified to be the most selective among ten anti-S100A8/S100A9 antibodies. In vitro, Ab45 could inhibit S100A8/S100A9-stimulated chemotaxis, and in vitro significantly reduced lung metastasis within a melanoma model [236]. The anti-S100P antibody 2H8 was introduced to inhibit S100P-induced proliferation of pancreatic cancer cells and blocked increased survival induced by S100P after gemcitabine treatment. Moreover, it was suggested that 2H8 is more effective than the small molecule inhibitor cromolyn, as cromolyn has a more significant impact on only tumour growth, while 2H8 reduced tumour growth and additionally inhibited liver metastasis in vivo [237]. 

However, the development of S100 antibodies for cancer therapy is at its very beginning, and examples of other therapeutic antibodies showed that limited clinical efficacy, for example, due to lacking tumour tissue penetration, immune reactions, and antibody resistance [252], is an issue which might need to be overcome in the later stages of this process.

### 3.3. Micro RNA (miRNA) Mimics

MiRNAs are short, non-coding nucleotides, which regulate gene expression on the post-transcriptional level by binding to their target mRNA and thereby either repress translation or tag the mRNA for degradation [253]. In recent years, new technology was established, which exploits this natural process of gene silencing by using miRNA mimics, chemically synthesised double-stranded RNAs, which act as mature miRNA once transfected into the cell [254]. 

Several miRNAs were introduced to target the expression of S100 proteins. Among them, two miRNAs, namely miR-187-3p and miR-149-3p, were found to downregulate S100A4 expression. The treatment of HCC in vitro and in vivo with miR-187-3p resulted in reduced metastasis and EMT, while miR-149-3p could inhibit invasion and migration of bladder cancer cells [238,239]. Furthermore, miR-193a suppressed proliferation, invasion, migration, and angiogenesis in vitro and in vivo within lung cancer models by silencing S100A6 [240]. 

The expression of S100A7 could be downregulated by miR-26b-5p, leading to decreased proliferation, migration, and invasion of intrahepatic cholangiocarcinoma in vitro [241]. Similar effects were observed for miR-24, a miRNA targeting S100A8, which inhibited proliferation and invasion of laryngeal carcinoma cells [242]. Besides, it was shown that S100A8-silencing by miR-24 reduced proliferation but enhanced sensitivity to paclitaxel of endometrial carcinoma cells [243]. Another interesting approach demonstrated that miR-6884-5p targets the expression of S100A16, which resulted in reduced proliferation, EMT, and invasion of gastric cancer cells in vitro [244].

The use of miRNA mimics, however, is a relatively new strategy for cancer therapy, and appropriate delivery systems need to be optimised to achieve effective treatment in the clinical context [255].

## 4. Conclusions 

Research over the past decades could show that S100 proteins are highly involved in many different cellular processes and are essential players in pathophysiological mechanisms. S100 family members are widely expressed proteins in vertebrates, yet their way of functioning is strongly dependent on the tissue context. Several exciting and promising approaches were made to exploit the current knowledge and use S100 proteins as valuable associates in the context of cancer therapy. However, far more research is needed to broadly establish S100 proteins as reliable biomarkers and to identify and further optimise safe and efficient S100 therapeutics. Given initial promising results, a better understanding of S100 proteins and their properties will bring great benefit to novel clinical applications.

## Figures and Tables

**Table 1:** Overview of potential S100 inhibitors.

Inhibitor	S100 Target	Mechanism of Action	Current Status	References
**Small Molecule Inhibitors**				
Calcimycin	Transcription of S100A4	Inhibition of β-catenin expression, which inhibits Wnt/β-catenin signalling and therefore expression of S100A4	Pre-clinical	[214]
Sulindac	Transcription of S100A4	Inhibition of β-catenin expression, which inhibits Wnt/β-catenin signalling and therefore expression of S100A4	Pre-clinical	[215]
Niclosamide	Transcription of S100A4	Inhibition of β-catenin/TCF complex binding to the S100A4 promoter leading to reduced expression of S100A4	Phase II for treatment of CRC metastasised patients	[216,217]
Trifluoperazine (TFP)	S100A4	Ca^2+^-S100A4/TFP dimers form a pentameric ring, disabling interaction between S100A4 and myosinIIA	Pre-clinical	[218,219]
Tasqinimod	S100A9	Binds S100A9 and blocks interaction with RAGE and TLR4	Phase III completed for treatment of metastatic castration-resistant prostate cancer (mCRPC)	[220,221,222,223]
1,2,4-triazoles	S100A10	Competes with annexin A2 for the binding to S100A10	Pre-clinical	[224,225]
Pentamidine	S100B	Binds to S100B and inhibits interaction with p53, therefore restoring p53 activity	Phase II for treatment of refractory melanoma	[226,227,228]
Cromolyn 5-methyl cromolyn	S100P	Binds to S100P and prevents interaction with RAGE	Pre-clinical	[229,230,231]
**Antibodies**				
6B12	S100A4	Binds extracellular S100A4 and thereby acts as an immunomodulating agent	Pre-clinical	[232,233]
5C3	S100A4	Binds to and neutralises S100A4	Pre-clinical	[234]
6F5	S100A7	Binds to S100A7 and thereby blocks S100A7/RAGE interaction	Pre-clinical	[235]
Ab45	S100A8/S100A9	Binds extracellular S100A8/S100A9 heterodimer and therefore reduces metastasis	Pre-clinical	[236]
2H8	S100P	Binds S100P and therefore reduces tumour growth and metastasis	Pre-clinical	[237]
**miRNA**				
miR-187-3p	S100A4	S100A4 silencing results in reduced metastasis and EMT in HCC	Pre-clinical	[238]
miR-149-3p	S100A4	S100A4 silencing results in inhibition of invasion and migration in bladder cancer cells	Pre-clinical	[239]
miR-193a	S100A6	S100A6 silencing results in suppression of proliferation, invasion, migration, and angiogenesis within lung cancer models	Pre-clinical	[240]
miR-26b-5p	S100A7	S100A7 silencing inhibits proliferation, invasion, and migration of intrahepatic cholangiocarcinoma cells	Pre-clinical	[241]
miR-24	S100A8	S100A8 silencing leads to decreased proliferation and invasion of laryngeal carcinoma cells and increases sensitivity of endometrial carcinoma cells for paclitaxel	Pre-clinical	[242,243]
miR-6884-5p	S100A16	S100A16 silencing reduces proliferation, invasion, and EMT in gastric cancer	Pre-clinical	[244]

## Abbreviations

AD	Alzheimer’s disease
ADC	adenocarcinoma
AMP	antimicrobial peptide
ANT	adenine nucleotide translocator
ATP	adenosine triphosphate
Bcl-2	namely B-cell lymphoma 2
Bcl-xL	B-cell lymphoma extra-large
Ca^2+^	calcium ion
Cacy/SIP	calcyclin-binding protein/Siah-1-interacting protein
CCL2	chemokine (C-C motif) ligand 2
COP9	constitutive photomorphogenesis 9
COPS5	COP9 constitutive photomorphogenic homolog subunit 5
COX2	cyclooxygenase-2
CP	calprotectin
CRC	colorectal carcinoma
CT	computerised tomography
Cu^2+^	copper ion
CXCL	chemokine (C-X-C motif) ligand
DAMPs	danger-associated molecular patterns DAMPs
ECG	electrocardiogram
ECM	extracellular matrix
EDC	epidermal differentiation complex
EGF	epidermal growth factor
EMMPRIN	extracellular matrix metalloprotease inducer
EMT	epithelial-mesenchymal transition
ERK	extracellular signal-regulated kinase
ERα	in estrogen receptor α
FABP	fatty acid-binding proteins
FAK	focal adhesion kinase
FDA	Food and Drug Administration
Fe^2+^	iron ion
HCC	hepatocellular carcinoma
HIF-1	hypoxia-inducible factor 1
IBD	inflammatory bowel disease
ICI	intracranial injuries
IL-1β	interleukin-1β
IFN-γ	interferon gamma
Jab1	c-Jun activation domain-binding protein-1
MAPK	mitogen-activated protein kinase
MAP3K8	mitogen-activated protein kinase kinase kinase 8
MCAM	melanoma cell adhesion molecule
MDR1	multidrug resistance gene 1
Mef2	myocyte enhancer factor-2
MRP1	MDR-associated protein 1
miRNA	micro RNA
MMP	metalloproteinase
Mn^2+^	manganese ion
mTOR	mechanistic target of rapamycin
NCX1	Na^+^/Ca^2+^ exchanger
Ndr	nuclear dbf2-related
NF-kB	nuclear factor ’kappa-light-chain-enhancer’ of activated B-cells
NMIBC	non-muscle-invasive bladder cancer
NMIIA	non-muscle myosin heavy chain IIA
NOXA	phorbol-12-myristate-13-acetate-induced protein 1
NSCLC	non-small cell lung cancer
OS	overall survival
OCSC	ovarian cancer stem cells
PAD3	phytoalexin deficient 3
PDGF	platelet-derived growth factor
PI3K	phosphoinositide 3-kinase
PMCA1b	plasma membrane Ca^2+^ transport ATPase
PUMA	p53 upregulated modulator of apoptosis
RA	rheumatoid arthritis
RAGE	receptor for advanced glycation end products
ROS	reactive oxygen species
RSK	ribosomal S6 kinase
RyR2	ryanodine receptor 2
SCC	squamous-cell carcinoma
SCLC	small cell lung cancer
SERCA2a	sarcoplasmic reticulum ATPase
Shh	sonic hedgehog
SNC	Scandinavian Neurotrauma Committee
STEMI	ST-segment elevation myocardial infarction
TAMs	tumour-associated macrophages
TBA	traumatic brain injury
TCF	T-cell factor
TLR4	Toll-like receptor 4
TME	tumour microenvironment
TNFα	tumour necrosis factor α
tPA	tissue plasminogen activator
TPL2	tumour progression locus 2
TRAF	TNF receptor-associated factor 2
TRAIL	tumour necrosis factor-related apoptosis-inducing ligand
TRPV6	transient receptor potential vanilloid 6
VEGF	vascular endothelial growth factor
YAP	yes-associated protein
Zn^2+^	zinc ion

## Appendix A

**Table A1:** Summary of the S100 family members.

Family Member	Expression	Regulation	Targets/Interaction	Function	References
S100A1	Cardiomyocytes, skeletal muscle cells, endothelial cells, neurological cells	Transcription factor binding sites for Nkx 2.5, Mef2 and CEF	SERCA2a/PLB, RyR2, F_1_-ATPase, ANT, Titin	Modulates contractile performance in cardiomyocytes: interaction with SERCA2a/PLB and RyR2 improves systolic Ca^2+^ release and diastolic Ca^2+^ uptake; binding to the PEVK subdomain of titin results in improved sarcomeric compliance Mitochondrial metabolism: plays a role in cardiac energy homeostasis by interacting with F_1_-ATPase and ANT	[36,37,256]
S100A2 (S100L; CaN19)	Epithelial tissue	Transcriptional activation by p53 EGF-induced transcription in keratinocytes	p53, RAGE	Tumour suppressor: calcium-dependent activation of p53	[51,78]
S100A3 (S100E)	Hair cuticular cells	PAD3 induces the formation of a homotetramer via citrullination	RARα	Maintenance of the hair texture: Ca^2+^-dependent epithelial barrier formation and cuticular maturation	[22,257,258]
S100A4 (metastasin; CAPL; calvasculin)	Fibroblasts, immune cells, cancer cells	Transcription is promoted by β-catenin and growth factors	actin, NMIIA, tropomyosin, p53, p37, MMP2, liprin-β1, RAGE, TLR4, EGFR, IL-10 receptor, annexin II	Cell migration: interaction with proteins of the cytoskeleton and induces secretion of matrix metalloproteinases (MMPs) Cell growth and proliferation: activates NF-κB and MAP kinases	[45,52,89]
S100A5	olfactory bulb, brainstem, spinal trigeminal tract	-	RAGE, NCXI	-	[34,259]
S100A6 (Calcylin; Cacy; CABP; PRA)	Fibroblasts, epithelial cells	Extracellular by PDGF, EGF, retonic acid, estrogen, gastrin Under stress conditions: ischemia, irradiation, oxidative stress Intracellular: NF-κB activates S100A6-promotor, while p53 indirectly suppresses transcription	caldesmon, calponin, tropomyosin, kinesin light chain, RAGE	Control of cell cycle progression: involved in ubiquitination of β-catenin Regulation of microfilament dynamics: interaction with proteins of the cytoskeleton Neuronal apoptosis: binding to RAGE leads to ROS-dependent activation of JNK, caspase-3, and caspase-7 As extracellular factor: involved in the release of lactogen II, insulin, and histamine	[27,260]
S100A7 (Psoriasin)	Keratinocytes	Secretion induced by disruptive stimuli (e.g., UVB, irradiation, retonic acid, infection with bacteria) Upregulated by pro-inflammatory cytokines (e.g., IL-1α, TNFα, IL-17, IL-22)	RAGE, FABP, TGM	Role as AMP: lyses bacteria (e.g., *E. coli*) Inhibition of epidermal differentiation: RAGE-dependent activation of NF-κB-signaling results in upregulation of IL-6 Inflammation: selective chemotactic for CD4^+^ lymphocytes and neutrophils	[28,42]
S100A8 (Calgranulin A)	Neutrophils, osteoclasts, hypertrophic chondrocytes, myeloid dendric cells	Induced by LPS, IL-10, TLR4, glucocorticoids	Telomerase	Inflammation: chemotactic for neutrophils, protects from oxidation by scavenging oxidants, acts as NO shuttle, thereby activating mast cells Regulation of differentiation: binds and inhibits telomerase, therefore promoting differentiation in keratinocytes	[43,261]
S100A9 (Calgranulin B)	Neutrophil granulocytes, monocytes	Upregulated by glucocorticoids, cytokines, and growth factors	RAGE, TLR4	Inflammation: Zn^2+^-dependent interaction with RAGE and TLR4 leads to upregulation of pro-inflammatory cytokines (e.g., TNFα) via NF-κB signalling; acts as a chemoattractant and is involved in differentiation of myeloid cells	[21,40,43]
S100A8/S100A9 (Calprotectin)	Neutrophils, monocytes, macrophages, inducible expression in keratinocytes and epithelial cells (e.g., under inflammatory conditions)	TNFα, IL-1β, HIF-1 stimulate expression Secretion is Ca^2+^-dependent	RAGE, TLR4	Modulation of the cytoskeleton: interacts with several proteins of the cytoskeleton (e.g., keratin and F-actin) and promotes polymerisation of microtubules Protection against pathogens: S100A8/S100A9 expressing epithelial cells are more resistant to bacterial infection Inflammation: RAGE- and TLR4-mediated upregulation of pro-inflammatory cytokines (e.g., IL-6 and IL-8) and adhesion proteins (e.g., ICAM-1)	[29]
S100A10 (p11)	Endothelial cells, macrophages, fibroblasts, epithelial cells	The expression can be induced by: Sp1, IFN-γ, glucocorticoids, TGFβ, EGF, IL-1β, thrombin, oncogenes (e.g., PML-RARα, KRas)	Annexin II, 5-HT1B receptor, TRPV5, TRPV6, TASK-1	Role as plasminogen receptor: regulates plasmin production by forming a complex with annexin II, which leads to fibrinolysis, activation of MMPs, ECM degradation, stimulation of the JAK1/TYK2 signalling pathway, and recruitment of macrophages Trafficking of plasma membrane proteins: S100A10 interacts with the 5-HT1B receptor, TRPV5, TRPV6, and TASK-1 and participates in their trafficking	[13,35]
S100A11 (S100C; calgizzarin)	Chondrocytes, keratinocytes, fibroblasts, luteal cells	TGFβ and high extracellular Ca^2+^ concentrations stimulate expression and activity	RAGE, annexin I, nucleolin	Cell growth regulation: intracellular S100A11 inhibits cell growth by binding to nucleolin, which allows expression of p21, leading to inhibition of DNA synthesis; extracellular S100A11 promotes cell proliferation by RAGE-mediated transcription of EGF Interaction with RAGE: RAGE activates p38 MAPK kinase, which increases type X collagen	[49,50]
S100A12 (Calgranulin C; EN-RAGE)	Neutrophil granulocytes, monocytes, macrophages, early-stage differentiating epithelial and dendric cells	Upregulated by TNFα and LPS Secretion in neutrophils involves ROS and K^+^ exchange	RAGE, TLR4, CacyBP/SIP	Wnt signalling: binds to CacyBP/SIP and is part of the ubiquitinylation complex Inflammation: binding to TLR4 leads to activation and migration of monocytes and release of IL-1β, IL-6, and IL-8, RAGE-mediated NF-κB and MAPK signalling induces secretion of TNFα and IL-1β Inhibition of pathogens: antifungal and antibacterial activity via Zn^2+^ sequestration	[41,262,263,264]
S100A13	Leydig cells of testis, follicle cells of thyroid, smooth muscle, endothelial and epithelial cells	Induced by stress stimuli	RAGE, Syt1, SPHK1	Non-canonical secretion pathway: forms a copper-dependent multiprotein complex with Syt1 and SPHK and is thereby involved in the non-classical stress-dependent release of FGF-1, IL-1α, and prothymosin-α	[265,266,267]
S100A14	High expression in epithelial tissue, lower expression in mesenchymal tissue	Regulated by p53	RAGE, p53	Interaction with RAGE: binding in lower concentrations activates MAPK and NF-κB signalling, leading to cell proliferation; binding in high concentrations activates ROS production, resulting in apoptosis	[46,53]
S100A15 (S100A7A; koebnerisin)	Dendric cells, endothelial cells, vascular smooth muscle cells, peripheral nerves, keratinocytes	Upregulated by *E. coli* through TLR4 Transcription can be induced by IFN-γ, IL-1β, TNFα, and Th1 Co-expression with S100A7 in keratinocytes	GPCR	Epidermal cell maturation: upregulated in epidermal differentiation Initial immune response: functions as an antibacterial agent by reducing the survival of *E. coli* and other strains Inflammation: acts as a chemoattractant for leucocytes via GPCR	[23,268]
S100A16	Astrocytes, adipocytes	Ca^2+^ influences nuclear import/export	p53	Overexpression in preadipocytes resulted in increased proliferation and reduction in insulin-stimulated glucose uptake and Akt phosphorylation	[269,270]
S100B	Astrocytes, oligodendrocytes, Schwann cells, ependymal cells, melanocytes, adipocytes, chondrocytes	Secretion is regulated by IL-1β, extracellular Ca^2+^ and K^+^, inhibitors of gap junctions, antioxidants, lipopolysaccharide, and apomorphine p53 upregulates expression	Extracellular: RAGE, FGFR1 Intracellular: Ndr kinase, Src, Rac1, IQGAP1, p53	Cell migration: regulates F-actin-based cytoskeleton via Src, IQGAP1, and Rac1 Cell division: interaction with Ndr kinase leads to the promotion of PI3K/Akt signalling Role in tumour suppression: inhibits p53 activity and reduces p53 Proliferation: at higher concentrations, S100B blocks RAGE and stimulates FGFR1, leading to Ras/MEK/Erk-mediated proliferation; at low concentrations, S100B additionally stimulates RAGE-mediated p38 MAPK signalling, resulting in activation of the mitogenic program	[47,65,271]
100G (CaBP-9k)	Epithelial cells	Vitamin D-dependent in the intestine	-	Cellular Ca^2+^ homeostasis: regulates intracellular Ca^2+^ levels and prevents toxic concentrations	[33]
S100P	Epithelial cells, leucocytes,	Promoter has binding sites for SMAD, STAT/CREB, and SP/KLF During embryonic implantation: highly expressed in the trophoblastic layer of the embryo, and in the endometrium of the uterine wall	RAGE, IQGAP1, enzrin, NMIIA	Cell proliferation and survival: activates RAGE-mediated NF-κB signalling; interaction with IQGAP1 induces MAPK signalling cascade Cell migration: promotes interaction with F-actin (via enzrin) and reduces focal adhesion sites (via NMIIA)	[48,272]
S100Z	Leucocytes	-	-	-	[8]
